# Global regional, and national burden of type 2 diabetes attributable to dietary factors from 1990 to 2021

**DOI:** 10.1038/s41598-025-98022-y

**Published:** 2025-04-17

**Authors:** Diya Xie, Fangqin You, Cheng Li, Daosen Zhou, Lihang Yang, Fengmin Liu

**Affiliations:** 1 Department of General Surgery, Fuzhou First General Hospital Affiliated with Fujian Medical University, Fuzhou, 350009 Fujian China; 2 Department of Endocrinology, Fuzhou First General Hospital Affiliated with Fujian Medical University, Fuzhou, 350009 Fujian China

**Keywords:** Type 2 diabetes, Dietary risk factors, Global burden of disease study, Public health strategies, Health inequality, Diseases, Endocrinology, Health care, Health occupations, Risk factors

## Abstract

The study comprehensively assesses the global burden of type 2 diabetes (T2D) linked to dietary factors from 1990 to 2021. Data from the Global Burden of Disease Study 2021 (GBD 2021) was analyzed for T2D dietary risk factors across 204 countries, using age-standardized metrics and SDI quintiles, with joinpoint regression and other analytical methods. The results revealed a significant increase in the global burden of T2D, with a 1.92-fold rise in DALYs and a 1.28-fold rise in total mortality cases due to dietary risks. High intake of processed meat and sugar-sweetened beverages were identified as major contributors to the increasing burden, whereas the age-standardized mortality rates attributable to low vegetable intake declined over time. The study also emphasized the impact of age, period, and cohort effects on the incidence of T2D, along with the growing health disparity gaps between high and low SDI countries. The study emphasizes the importance of dietary interventions to reduce T2D burden, suggesting a decrease in processed meat and sugary drink consumption and an increase in vegetable intake. It calls for global strategies to address health disparities and improve equitable access to health resources.

**Supplementary Information:**

The online version contains supplementary material available at 10.1038/s41598-025-98022-y.

## Introduction

According to the latest data, the prevalence and number of patients with T2D globally continue to rise. Data from 2021 shows that there are 537 million (10.5%) diabetes patients among adults aged 20–79, affecting approximately one in ten adults^1^. Compared to 2019, there has been an increase of 74 million diabetes patients, representing a 16% rise and highlighting the alarming growth in global diabetes prevalence^1^. The International Diabetes Federation (IDF) estimates that by 2030, the number of diabetes patients will increase to 643 million (11.3%), and by 2045, it will rise to 783 million (12.2%), with a 46% increase, more than double the estimated population growth rate during the same period (20%). The proportion of adults affected by diabetes may reach one-eighth^1,2^. Furthermore, the health burden caused by diabetes is substantial. In 2021, global health expenditures due to diabetes were estimated at $966 billion, accounting for 9% of global health spending and increasing by approximately 316% over the past 15 years^3^. In the same year, diabetes or its complications led to around 6.7 million deaths among adults aged 20–79, representing over 12.2% of overall deaths in that age group^4^. Additionally, an estimated 240 million diabetes patients globally remain undiagnosed, with over 81% of them living in lower to middle incomes countries. In some regions including Africa, Southeast Asia, and the Western Pacific, over half of diabetes patients remain undiagnosed^5^.

The *Lancet* has recently published a result from the Global Burden of Disease Study 2021 (GBD 2021), estimating the global burden of diabetes from 1990 to 2021 and making predictions for 2050^6^. The analysis reveals that in 2021, there were 529 million diabetes patients globally, with 96.0% being T2D^7^. Among the population aged 20–79, an estimated 485 million individuals had diabetes. In 43 countries/regions worldwide, the age-standardized prevalence of diabetes exceeded 10%^8^. By 2050, it is projected that there will be 1.31 billion diabetes patients globally^6^. These figures underscore the challenge diabetes poses to the global public health system and the need for effective prevention and treatment strategies to address this increasingly serious health issue.

Further attribution analysis reveals that the majority of disability-adjusted life years (DALYs) attributed to T2D are due to risk factors^9^. High BMI and dietary risk factors are the primary risk factors for T2D^10^. Current research widely acknowledges the significant impact of dietary patterns on T2D. Diets with a low glycemic index, Mediterranean diet, and DASH diet, among others, have been shown to have a positive effect on improving glucose and lipids metabolism in patients with T2D^11–13^. Particularly, the Mediterranean diet, by increasing dietary fiber, n-3 fatty acids, and antioxidants intake, has been proven to reduce the risk of developing type 2 diabetes and aid in disease management^14,15^. Additionally, moderate intake of high-quality proteins such as fish, poultry, dairy, and legumes while limiting processed and red meat consumption can lower the risk of developing T2D^16^. Studies indicate that the consumption of vegetables and fruits is associated with a lower risk of developing T2D, especially those rich in antioxidants and fiber^17^. However, the specific burden of T2D caused by dietary risks has not been fully elucidated, and previous research has mainly focused on T2D, independent dietary factors, or the incidence rates in particular geographic areas and nations^18,19^.

The global burden of T2D is continuously increasing, necessitating targeted strategies to be promptly implemented, including changes in dietary patterns, increased physical activity, and lifestyle interventions^20^. This study offers a comprehensive analysis of how seven dietary risk factors contribute to the global landscape of T2D. It aims to enhance our understanding of how these factors impact mortality and disability across diverse populations and socioeconomic groups, examining trends from 1990 to 2021. By investigating these factors in 204 nations, 5 SDI regions and 21 global disease zones, the research fills critical gaps in current literature regarding the role of diet in T2D. The study aims to inform the development of targeted policies and interventions to address the public health impact of T2D on a global scale.

## Methods

### Data source

In the GBD 2021 study, data aggregation was conducted through a systematic review of surveys, censuses, civil registries, demographic surveillance, and various health data sources. After the compilation phase, each data source underwent a thorough bias assessment, and corrections were applied using standardized statistical estimation methods facilitated by the DisMod-MR 2.1 tool, which utilizes Bayesian meta-regression techniques. Detailed procedures for data collection, processing, and finalization can be found in the GBD 2021 project documentation^21,22^. Public estimates are accessible through the Global Health Data Exchange (GHDx) online platform^23^. SDI served as a crucial indicator of socioeconomic status closely associated with health outcomes. SDI is calculated as the geometric mean of three normalized metrics: under-25 fertility rates, educational attainment among individuals aged 15 and above, and time-adjusted per capita income, each ranging from 0 to 1. GBD 2021 classifies SDIs into five quintiles: high, high-middle, middle, low-middle, and low.

The diagnostic standard for T2D involves fasting plasma glucose exceeding 126 mg/dL, with additional indicators such as glucose tolerance tests also recognized^24^. These criteria were adjusted during the modeling phase. Our research investigated age-adjusted T2D burden data associated with dietary elements in adults spanning from 1990 to 2021, segmented by demographic groups. The dietary risk factors considered (Table S1) encompassed low intake of vegetables, whole grains, fiber, and fruits, as well as high consumption of processed meat, red meat, and sugar-sweetened beverages^23^. These dietary patterns were assessed through a 24-hour recall survey, quantifying daily food and nutrient intake per individual in grams. Our research spanned 204 nations, 21 worldwide disease zones, and 5 SDI regions, following the Guidelines for Accurate and Transparent Health Estimates Reporting (GATHER) guidelines for transparent health estimate reporting^25^. Metrics such as age-standardized mortality rates (ASMRs) and age-standardized disability-adjusted life-year rates (ASDRs) were calculated per 100,000 individuals, with 95% uncertainty intervals (95%UI) provided. The contribution of dietary risk factors to the burden of T2D was quantified using Population Attributable Fractions (PAFs), derived by comparing theoretical minimum risk levels with actual population exposures, assuming constancy in other risk factors. The attributable burden was determined by applying the relevant PAFs to the total T2D burden across age, gender, location, and year categories^22^.

### Joinpoint analysis

We analyzed temporal trends in T2D burden from 1990 to 2021 using Joinpoint regression (National Cancer Institute, Version 4.9.1.0), which identifies statistically significant inflection points (Joinpoints) where trend direction or magnitude changed^26^. The method segments time-series data into distinct phases through permutation testing, calculating Annual Percentage Change (APC) for each phase and an Average Annual Percentage Change (AAPC) as the geometric mean of APC values weighted by segment duration. For example, an AAPC of + 0.1 indicates a sustained 0.1% annual increase in age-standardized rates over the entire period. A confirmed joinpoint in 2008—where APC of mortality rates shifted from a + 3.0% annual increase (1990–2008) to a −0.5% decline (2009–2021)—would signal altered disease dynamics, potentially linked to policy interventions. We applied logarithmic-linear models ASDR and ASMR to account for Poisson-distributed outcomes, with technical details on Monte Carlo optimization and grid search algorithms provided in Supplementary Methods.

### Age-period-cohort model

We employed an age-period-cohort model to investigate the temporal dynamics of DALYs and mortality rates related to T2D. A weighted least squares regression approach was utilized to address the inherent collinearity among age, period, and cohort variables^27^. The model was customized to estimate four key indicators: net drift, capturing the overall annual percentage change in age-adjusted rates over time; local drift, illustrating the annual percentage change in age-specific rates; period relative risks (RRs), calculated by comparing each period’s age-standardized rate (ASR) with the reference year of 2002; and cohort relative risks (RRs), determined by contrasting each cohort’s ASR with a reference cohort adjusted for the reference age of 50 years^28^. Furthermore, longitudinal age curves were generated to display rates for each age group within a reference cohort, accounting for period variations. The data was segmented into consecutive 5-year age and period groups, as well as birth cohorts. The Wald chi-square test was utilized to evaluate the significance of the model’s estimated variables, providing a robust analytical framework to comprehend the intricate interplay of age, period, and cohort effects on T2D incidence and prevalence. The age-period-cohort model was executed using the National Cancer Institute’s age-period-cohort web tool^29^.

### Decomposition analysis

We conducted a three-factor decomposition analysis (population growth, age structure shifts, and composite dietary risk factors) using Das Gupta’s framework to quantify drivers of T2D burden changes (1990–2021). Age-standardized rates isolated demographic effects, while counterfactual scenarios sequentially fixed one factor (e.g., holding age structure constant) to partition total changes into additive contributions. The composite risk factor category encompassed seven modifiable dietary/metabolic risks analyzed collectively. Percentage contributions reflect each factor’s proportional impact on total burden changes, distinguishing unavoidable demographic trends from actionable risk exposures^30^. Methodological details are provided in Supplementary Methods.

### Cross-country inequality analysis

It is imperative to monitor health disparities to inform evidence-based health strategies, improve policies, and refine practices aimed at reducing inequalities in health outcomes^31^. In this study, two fundamental measures of health inequality, the slope index of inequality (SII) and the concentration index (CI), were utilized to evaluate the distribution of the burden of T2D across different nations^32^. The SII was calculated through a regression analysis of a country or territory’s total DALYs and mortality rates against SDI scale. The relative position on this scale was determined by the median of the population’s cumulative ranking based on SDI. To address data variance, a weighted regression approach was employed. The CI was determined by calculating the area under the Lorenz curve, which was constructed using the cumulative proportion of crude DALYs and mortality rates alongside the cumulative distribution of the population ranked by SDI. This method facilitated a measurable evaluation of the concentration of health outcomes across the sociodemographic spectrum. Statistical analyses were carried out using R software (version 4.4.1), with the “ggplot2” package utilized for data visualization. A significance level of *p* < 0.05 was considered statistically significant.

## Results

### Overall burden of T2D attributable to seven dietary risks

**Fig. 1:**
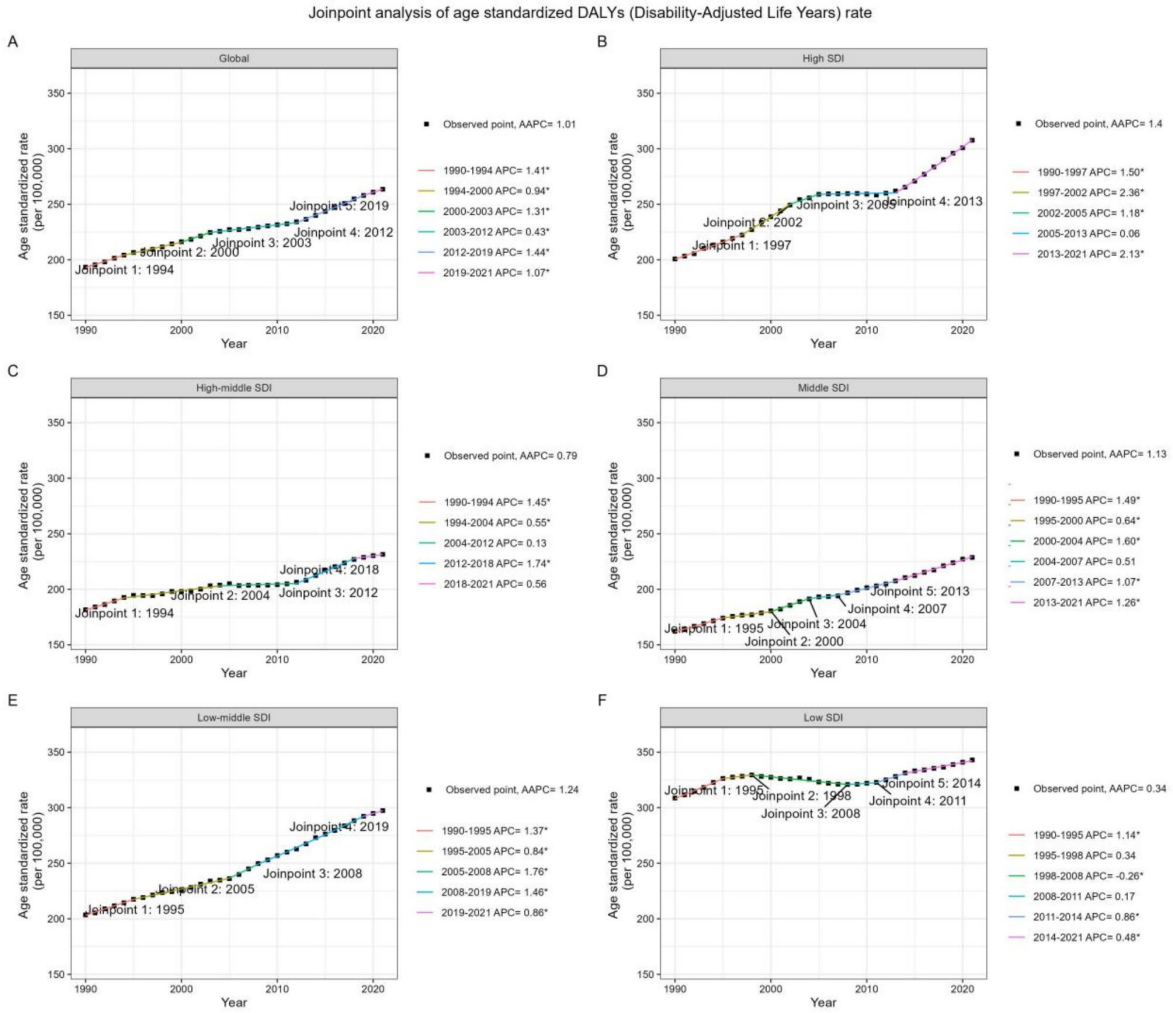
Global trends in ASDR for type 2 diabetes (T2D) attributable to dietary risk factors and across five SDI quintiles from 1990 to 2021. ASDR, age standardized disability-adjusted life-years rate; AAPC, average annual percent change; APC, annual percent change; SDI, Socio-Demographic Index.

**Fig. 2:**
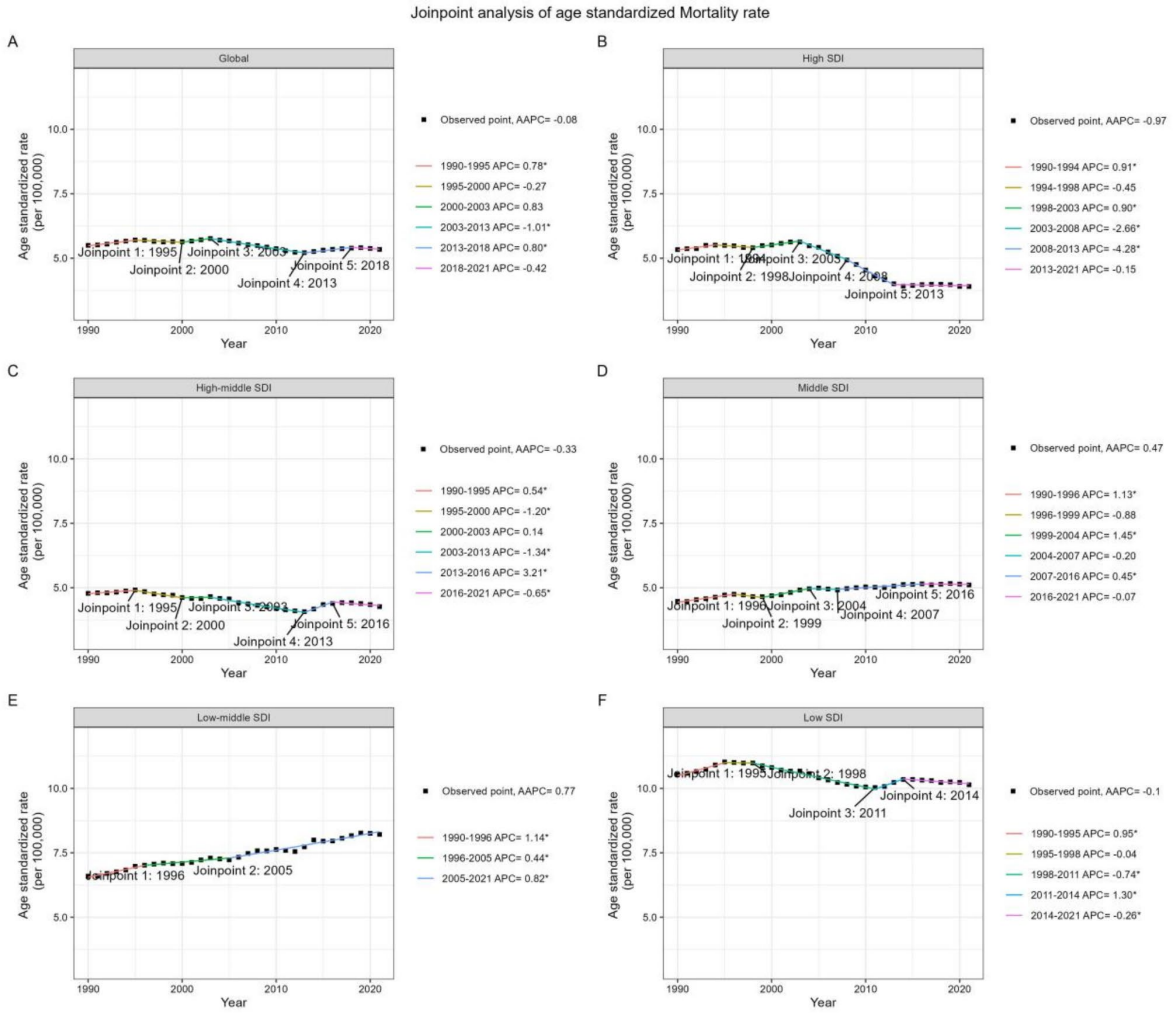
Global trends in ASMR for type 2 diabetes (T2D) attributable to dietary risk factors and across five SDI quintiles from 1990 to 2021. ASMR, age standardized mortality rate; AAPC, average annual percent change; APC, annual percent change; SDI, Socio-Demographic Index.

Globally, DALYs related to T2D due to dietary risks saw a rise from 7,798,781.99 (95%UI: 1,475,237.25–13,876,382.94) in 1990 to 22,785,311.29 (95%UI: 4,642,735.64–40,829,142.16) in 2021, marking a 1.92-fold increase (Table 1). The total mortality cases rose from 197,934.49 (95%UI: 36,223.06–344,570.61) in 1990 to 450,501.06 (95%UI: 87,925.80 to 783,806.52) in 2021, a 1.28-fold increase (Table 2). ARS for these measures showed divergent trends. ASDR increased from 193.39 (95%UI: 36.62–343.95) in 1990 to 263.50 (95%UI: 53.76–472.02) in 2021, with an AAPC of 1.01 (95% CI 0.94–1.08) (Fig. 1). Meanwhile, ASMR remained stable with an AAPC of −0.08 (95% CI −0.19 to 0.03), at 5.50 (95%UI: 1.02–9.56) in 1990 and 5.34 (95%UI: 1.04–9.29) in 2021 (Fig. 2). From 1990 to 2021, ASDR increased for both females and males, with AAPCs of 0.90 (95% CI 0.83–0.97) and 1.13 (95% CI 1.09–1.17) respectively. Female ASMR decreased with an AAPC of −0.21 (95% CI −0.37 to −0.06), while male ASMR remained stable with an AAPC of 0.08 (95% CI −0.04 to 0.20). The global ASDR for the entire population continuously rose over the 31 years, with the most recent acceleration seen after 2012. In contrast, global ASMR exhibited a fluctuating trend, with a significant decline from 2003 to 2013 (APC = −1.01, 95%CI −1.09 to −0.93), followed by an increase from 2013 to 2018 (APC = 0.80, 95%CI 0.52–1.09), and recent stability.

**Table 1:** Disability-Adjusted life years (DALYs) for type 2 diabetes (T2D) attributed to dietary risk factors: age-standardized rates with 95% uncertainty intervals and annual percentage change (AAPC) with 95% confidence intervals, 1990–2021.

	1990		2021		1990–2021
	DALYs (Disability-Adjusted Life Years) cases No. *10^5^ (95% UI)	ASDR per 100,000 No. (95% UI)	DALYs (Disability-Adjusted Life Years) cases No. *10^5^ (95% UI)	ASDR per 100,000 No. (95% UI)	AAPC No. (95% CI)
Global	77.99 [14.75–138.76]	193.39 [36.62–343.95]	227.85 [46.43–408.29]	263.50 [53.76–472.02]	1.01 [0.94–1.08]
Female	40.18 [7.62–71.30]	187.36 [35.59–332.12]	111.88 [22.93–199.58]	246.81 [50.71–440.22]	0.90 [0.83–0.97]
Male	37.81 [7.10–67.35]	200.02 [37.52–357.42]	115.97 [23.35–208.54]	281.86 [56.83–506.44]	1.13 [1.09–1.17]
High SDI	21.74 [4.47–38.47]	200.76 [41.41–355.24]	56.49 [12.31–102.04]	307.60 [67.32–557.20]	1.40 [1.31–1.48]
High middle SDI	18.11 [3.76–32.23]	181.56 [37.93–322.46]	44.26 [8.89–80.18]	231.41 [46.65–418.72]	0.79 [0.69–0.89]
Middle SDI	17.84 [3.44–32.26]	162.24 [30.94–293.64]	63.24 [13.19–114.06]	228.67 [47.63–412.46]	1.13 [1.04–1.22]
Low middle SDI	12.82 [2.21–22.79]	203.51 [34.37–362.53]	44.59 [9.25–78.67]	297.39 [60.98–525.52]	1.24 [1.14–1.33]
Low SDI	7.35 [0.73–13.25]	308.73 [29.78–555.47]	19.01 [2.41–34.32]	343.01 [42.41–620.69]	0.34 [0.23–0.45]

DALYs, disability-adjusted life years; ASDR, age standardized disability-adjusted life years rate; AAPC, average annual percent change; SDI, Socio-Demographic Index; UI, uncertainty interval; 95% CI, 95% confidential intervals.

**Table 2:** Mortality for type 2 diabetes (T2D) attributed to dietary risk factors: age-standardized rates with 95% uncertainty intervals and annual percentage change (AAPC) with 95% confidence intervals, 1990–2021.

	1990		2021		1990–2021
	Mortality cases No. *10^5^ (95% UI)	ASMR per 100,000 No. (95% UI)	Mortality cases No. *10^5^ (95% UI)	ASMR per 100,000 No. (95% UI)	AAPC No. (95% CI)
Global	1.98 [0.36–3.45]	5.50 [1.02–9.56]	4.51 [0.88–7.84]	5.34 [1.04–9.29]	−0.08 [−0.19 to 0.03]
Female	1.09 [0.20–1.91]	5.41 [1.01–9.44]	2.34 [0.47–4.08]	5.03 [1.00–8.77]	−0.21 [−0.37 to −0.06]
Male	0.88 [0.16–1.55]	5.60 [1.02–9.79]	2.17 [0.42–3.78]	5.72 [1.10–9.99]	0.08 [−0.04 to 0.20]
High SDI	0.59 [0.12–1.03]	5.33 [1.08–9.22]	0.88 [0.19–1.52]	3.90 [0.85–6.72]	−0.97 [−1.23 to −0.71]
High middle SDI	0.43 [0.09–0.74]	4.78 [1.02–8.23]	0.83 [0.17–1.44]	4.27 [0.89–7.37]	−0.33 [−0.56 to −0.10]
Middle SDI	0.40 [0.08–0.71]	4.47 [0.83–7.94]	1.30 [0.27–2.30]	5.11 [1.04–9.05]	0.47 [0.24 to 0.70]
Low middle SDI	0.34 [0.06–0.60]	6.59 [1.06–11.72]	1.05 [0.20–1.80]	8.21 [1.54–14.21]	0.77 [0.65 to 0.90]
Low SDI	0.21 [0.02–0.38]	10.53 [0.94–19.02]	0.44 [0.05–0.80]	10.14 [1.15–18.25]	−0.10 [−0.25 to 0.05]

ASMR, age standardized mortality rate; AAPC, average annual percent change; SDI, Socio-Demographic Index; UI, uncertainty interval; 95% CI, 95% confidential intervals.

When analyzing ASDR across SDI quintiles, the lowest SDI category maintained the highest rates in both 1990 and 2021, with minimal change noted (AAPC = 0.34, 95% CI 0.23–0.45). In contrast, the high SDI quintile showed the most rapid change, ascending from the third rank in 1990 to the second in 2021 (AAPC = 1.40, 95% CI 1.31–1.48). ASDR increased across 21 regions, except for Eastern Sub-Saharan Africa, which notably decreased (AAPC = −0.80, 95% CI −0.90 to −0.71) (Table S2). Central Asia experienced the most substantial rise in ASDR over the study period (AAPC = 2.25, 95% CI 2.00–2.51). Regarding ASMR, the low SDI category remained stable over the 31-year period, maintaining the highest rates. Conversely, the high SDI category declined from the third rank in 1990 to the lowest rank in 2021 (AAPC = −0.97, 95% CI −1.23 to −0.71) (Table S3). The high-middle SDI category also decreased, while the middle and low-middle SDI categories saw increases over the 31-year period. Eastern Europe experienced the fastest increase in ASMR, while the High-income Asia Pacific region witnessed the most rapid decline.

In 2021, Fiji and the Marshall Islands topped the list for both ASDR and ASMR, with Samoa ranking third in ASDR and Kiribati third in ASMR. Over the 31-year period, Guatemala (AAPC = 4.31, 95% CI 3.67–4.96), Morocco (AAPC = 3.13, 95% CI 3.05–3.21), and Uzbekistan (AAPC = 3.11, 95% CI 2.38–3.84) had the highest AAPC values for ASDR. Similarly, for ASMR, Guatemala (AAPC = 4.28, 95% CI 3.56–5.01), the Russian Federation (AAPC = 4.24, 95% CI 3.54–4.94), and Cabo Verde (AAPC = 3.53, 95% CI 2.79–4.27) showed significant AAPC values. Notably, Singapore witnessed a remarkable decline in ASMR (AAPC = −6.21, 95% CI −7.03 to −5.37), while Rwanda saw a comparable reduction in ASDR (AAPC = −2.33, 95% CI −2.52 to −2.15).

### Burden of T2D attributable to each of seven dietary risks

Globally, the T2D ASDR attributable to dietary risks increased for most dietary risks, although the rate linked to low vegetable intake declined (AAPC = −1.13, 95% CI −1.22 to −1.04) (Table 3). High processed meat intake emerged as a critical driver of ASDR growth (global AAPC = 1.07), with low-middle SDI countries experiencing the fastest acceleration (AAPC = 2.12) and East Asia showing the highest regional burden increase (AAPC = 3.27). In contrast, the global T2D ASMR for processed meat intake declined (AAPC=−0.28), masking stark regional divergence: high-SDI nations achieved significant reductions (AAPC=−1.15), while lower SDI regions continued to face rising ASMR burdens.

**Table 3:** DALYs and mortality for type 2 diabetes (T2D) attributed to each dietary risk factors: numbers and age-standardized rates with 95% uncertainty intervals in 2021 and annual percentage change (AAPC) with 95% confidence intervals from 1990 to 2021.

	2021		1990–2021	2021		1990–2021
	DALYs (Disability-Adjusted Life Years) cases No. *10^5^ (95% UI)	ASDR per 100,000 No. (95% UI)	AAPC of ASDR No. (95% CI)	Mortality Cases No. *10^5^ (95% UI)	ASMR per 100,000 No. (95% UI)	AAPC of ASMR No. (95% CI)
Diet high in processed meat	61.03 [14.79–104.38]	70.58 [17.11–120.71]	1.07 [1.01–1.14]	1.15 [0.27–1.91]	1.37 [0.32–2.27]	−0.28 [−0.43 to −0.13]
Diet high in red meat	38.25 [−5.85 to 87.13]	44.10 [−6.73 to 100.38]	1.38 [1.29–1.48]	0.69 [−0.10 to 1.53]	0.82 [−0.12 to 1.81]	0.14 [−0.03 to 0.31]
Diet high in sugar sweetened beverages	30.29 [15.22–45.92]	35.02 [17.59–53.13]	2.23 [2.14–2.31]	0.54 [0.28–0.79]	0.64 [0.33–0.93]	0.86 [0.69–1.04]
Diet low in fiber	7.75 [4.27–11.58]	9.02 [4.97–13.46]	0.27 [0.22–0.32]	0.17 [0.10–0.25]	0.21 [0.12–0.30]	−0.52 [−0.65 to −0.38]
Diet low in fruits	33.81 [5.32–60.76]	39.14 [6.17–70.26]	0.50 [0.43–0.58]	0.77 [0.12–1.35]	0.91 [0.14–1.59]	0.04 [−0.11 to 0.19]
Diet low in vegetables	5.91 [−2.28 to 13.08]	6.87 [−2.66 to 15.21]	−1.13 [−1.22 to −1.04]	0.16 [−0.06 to 0.36]	0.19 [−0.07 to 0.42]	−1.61 [−1.73 to −1.50]
Diet low in whole grains	50.81 [14.96–85.44]	58.78 [17.32–98.86]	0.93 [0.90–0.96]	1.02 [0.28–1.66]	1.21 [0.33–1.98]	−0.09 [−0.21 to 0.03]
All dietary risks	227.85 [46.43–408.29]	263.50 [53.76–472.02]	1.01 [0.94–1.08]	4.51 [0.88–7.84]	5.34 [1.04–9.29]	−0.08 [−0.19 to 0.03]

DALYs, disability-adjusted life years; ASDR, age standardized disability-adjusted life years rate; ASMR, age standardized mortality rate; AAPC, average annual percent change; SDI, Socio-Demographic Index; UI, uncertainty interval; 95% CI, 95% confidential intervals.

Red meat consumption exhibited dual burdens: while its global ASDR impact escalated (AAPC = 1.38), driven by Southeast Asia (AAPC = 3.26) and East Asia (AAPC = 2.71), the ASMR consequences disproportionately affected Eastern Europe (AAPC = 2.93) and South Asia (AAPC = 2.83). Similarly, low whole grain intake demonstrated divergent trajectories—its ASDR burden grew fastest in high-income Asia Pacific (AAPC = 2.69), whereas ASMR impacts were most severe in Eastern Europe (AAPC = 2.63), despite Southern Latin America achieving notable ASMR reductions (AAPC=−2.21).

The T2D ASDR attributable to high sugar-sweetened beverage consumption surged globally (AAPC = 2.23, 95% CI 2.14–2.31), peaking in East Asia (AAPC = 5.64) and Eastern Europe (AAPC = 3.87). This contrasted sharply with ASMR patterns, where Eastern Europe recorded the most alarming mortality increase (AAPC = 5.33), despite global ASMR growth being comparatively modest (AAPC = 0.86).

SDI-based stratification revealed fundamental disparities: high-SDI countries faced sustained ASDR threats from sugar-sweetened beverages (AAPC = 2.44) and insufficient whole grains (AAPC = 1.55), yet achieved universal ASMR declines across all risk factors. Conversely, low-SDI nations struggled with concurrent ASDR increases from processed meat (AAPC = 0.84), red meat (AAPC = 0.85), and sugar-sweetened beverages (AAPC = 1.37), though their ASMR for low vegetable intake showed marked improvement (AAPC=−0.96).

Regionally, East Asia’s dual burden of rapid industrialization and dietary transition was evident: it led global ASDR growth for sugar-sweetened beverages (AAPC = 5.64) and processed meats (AAPC = 3.27), yet simultaneously achieved the sharpest ASDR decline for low vegetable intake (AAPC=−5.68). Eastern Europe presented a unique challenge, with uniformly rising ASDR and ASMR across multiple risks—most critically for sugar-sweetened beverages (ASDR AAPC = 3.87; ASMR AAPC = 5.33) and low fiber intake (ASDR AAPC = 3.00; ASMR AAPC = 4.46) .

**Fig. 3:**
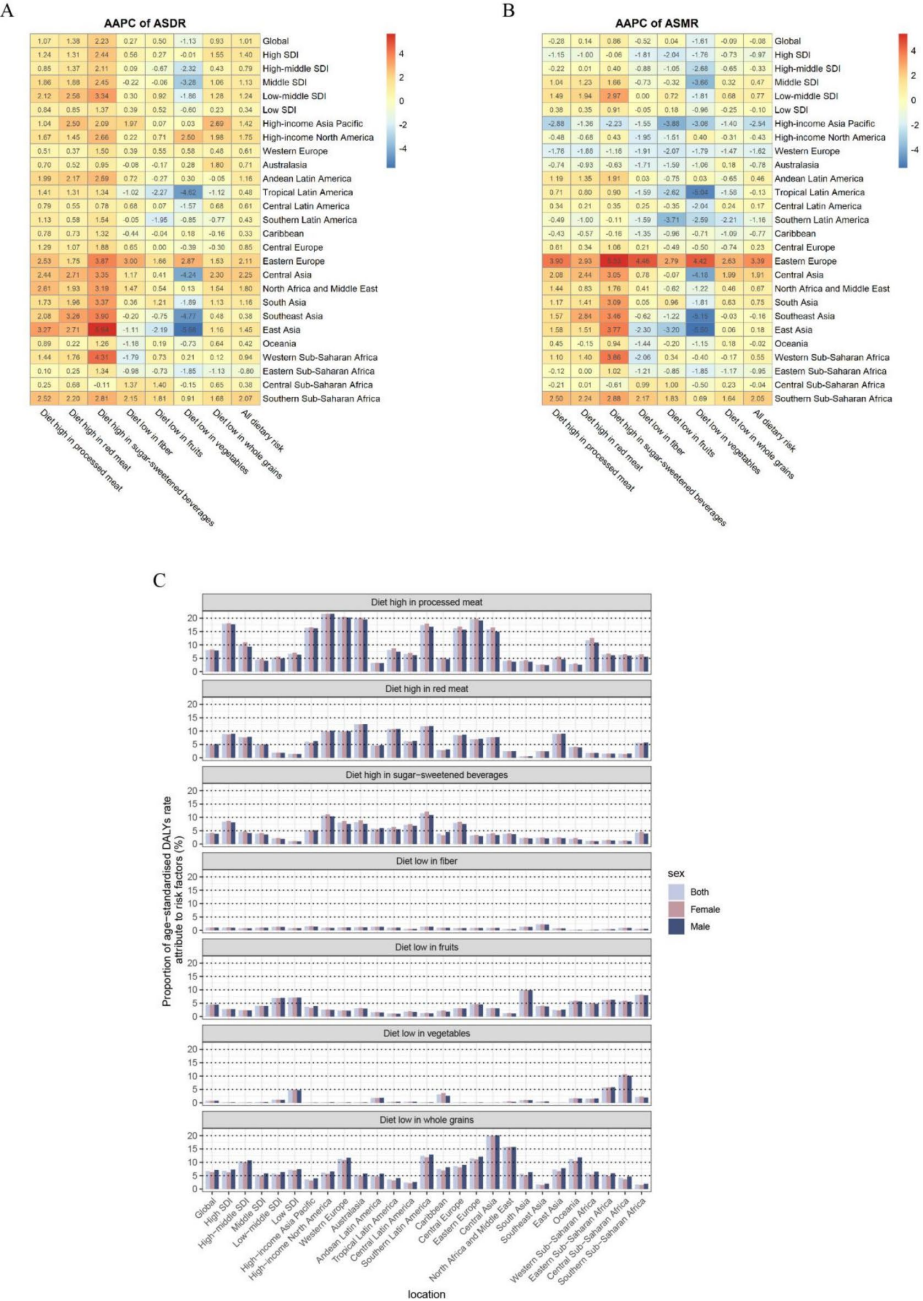
Global trends in the burden of Type 2 Diabetes (T2D) attributable to each dietary risk factors across five Socio-Demographic Index (SDI) quintiles and 21 regions from 1990 to 2021. (**A**) Average annual percent change (AAPC) in age-standardized disability-adjusted life-years (DALY) rate due to T2D from 1990 to 2021. (**B**) AAPC in age-standardized mortality rate (ASMR) due to T2D from 1990 to 2021. (**C**) Population attributable fractions for T2D related to each dietary risk factors in 2021. DALYs, disability-adjusted life-years; ASDR, age standardized disability-adjusted life-years rate; ASMR, age standardized mortality rate; AAPC, average annual percent change; APC, annual percent change; SDI, Socio-Demographic Index; PAF, population attributable fractions.

Low fruit intake exemplified geographic specificity: Southern Sub-Saharan Africa bore the highest ASDR increase (AAPC = 1.86), while Eastern Europe suffered the greatest ASMR escalation (AAPC = 2.79). High-income Asia Pacific and Western Europe stood out with consistent ASMR reductions across all seven risk factors, underscoring the effectiveness of integrated nutritional policies in these regions (Fig. 3A and B, Table S4 and S5).

### PAF of each dietary risk factor

In 2021, high processed meat consumption globally had the highest PAF for ASDR related to T2D, at 8.07% (95% CI 1.96–13.45) (Fig. 3C, Table S6). In contrast, PAFs for low fiber and vegetable intake were notably lower, at 1.03% (95% CI 0.59–1.47) and 0.79% (95% CI −0.29 to 1.71), respectively. When examining the five SDI quintiles, a consistent upward trend in PAF was observed for specific dietary factors, particularly diets high in processed and red meats. This trend was most pronounced in the highest SDI quintile, where the PAF for processed meat intake was 17.87% (95% CI 4.39–28.83), compared to 6.68% (95% CI 1.59–11.30) in the lowest quintile.

Regional analysis revealed diverse patterns. Sub-Saharan Africa showed a PAF of 3.16% (95% CI −1.25 to 6.98) for low vegetable intake, notably higher than the 0.02% (95% CI −0.01 to 0.05) in East Asia. High intakes of processed and red meats, along with low intake of whole grains, were the most impactful factors on T2D burden, with PAFs exceeding 20% in some regions. Conversely, low fiber and fruit intake had a more consistent but generally lower impact, reflecting a global dietary trend. Modest gender differences in PAF suggest a uniform influence of dietary risk factors on T2D across genders.

### Age-period-cohort model

**Fig. 4:**
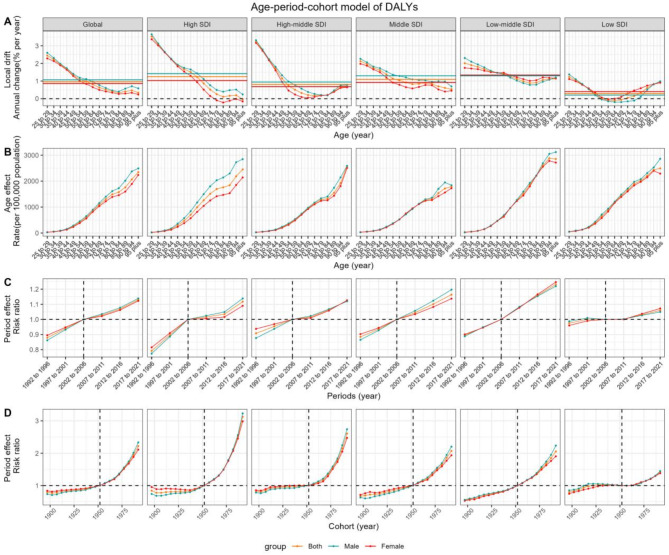
Estimates of age, period, and cohort effects on disability-adjusted life-years (DALYs) due to Type 2 Diabetes (T2D) attributed to dietary risk factors globally and in different SDI regions. (**A**) Local drift, the horizontal-colored lines represent the net drift of different groups; (**B**) Age effect; (**C**) Period effect; (**D**) Cohort effect. T2D, type 2 diabetes; SDI, socio-demographic index; DALYs, disability-adjusted life-years.

**Fig. 5:**
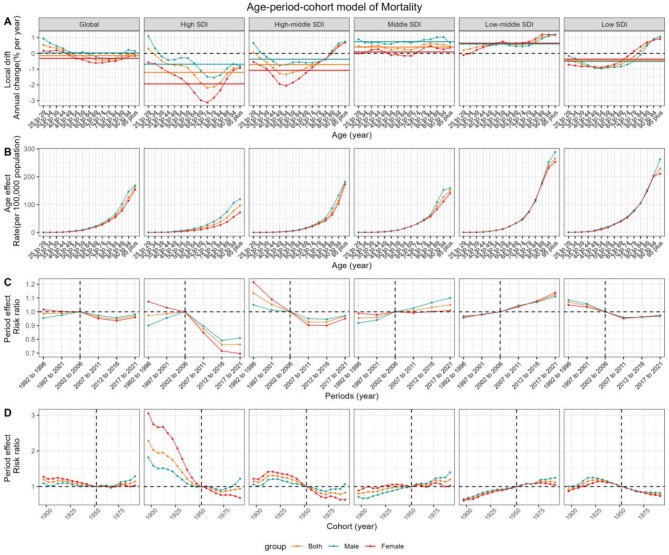
Estimates of age, period, and cohort effects on mortality due to Type 2 Diabetes (T2D) attributed to dietary risk factors globally and in different SDI regions. (**A**) Local drift, the horizontal-colored lines represent the net drift of different groups; (**B**) Age effect; (**C**) Period effect; (**D**) Cohort effect. T2D, type 2 diabetes; SDI, socio-demographic index; DALYs, disability-adjusted life-years.

In our analysis using the APC model (Figs. 4 and 5), we explored the impact of age, period, and cohort on the load of T2D attributed to dietary risk elements. The net drift in DALYs was consistently positive across all SDI quintiles, aligning with our previous findings of increasing DALYs over the 31-year period. The local drift in DALYs followed a U-shaped pattern, with the minimum point shifting towards older age categories as SDI increased. Although the overall trend indicated an increase in DALYs, the rate of increase was less pronounced among the middle-aged group compared to other age groups. Males in the middle-aged to elderly categories showed a higher local drift in DALYs than females in high, high-middle, and middle SDI quintiles, but lower values compared to females in low-middle and low SDI quintiles. In terms of mortality trends, the net drift was negative in the high, high-middle, and low SDI quintiles, while it was positive in the middle and low-middle quintiles. Notably, the lowest mortality local drift was observed among the 70–79-year-old population in the high SDI quintile.

In terms of period effects, RRs for DALYs increased over the period across all SDI quintiles, with a more subdued rise in the lowest quintile. Conversely, period RRs for mortality decreased in the high, high-middle, and low SDI quintiles, while they increased in the middle and low-middle quintiles. The high SDI quintile experienced the most significant decline, with females showing a greater reduction than males.

Regarding cohort effects, RRs for DALYs increased with successive birth cohorts, with the highest change observed in the high SDI quintile and the least in the low SDI quintile. For mortality, cohort RRs decreased in the high, high-middle, and low SDI quintiles after 1912, while they continued to rise in the middle and low-middle quintiles. The high SDI quintile experienced the most significant decline in cohort RRs for mortality. However, a concerning upward trend in mortality RRs was noted among males in the most recent cohorts across most SDI quintiles.

### Decomposition analysis

**Fig. 6:**
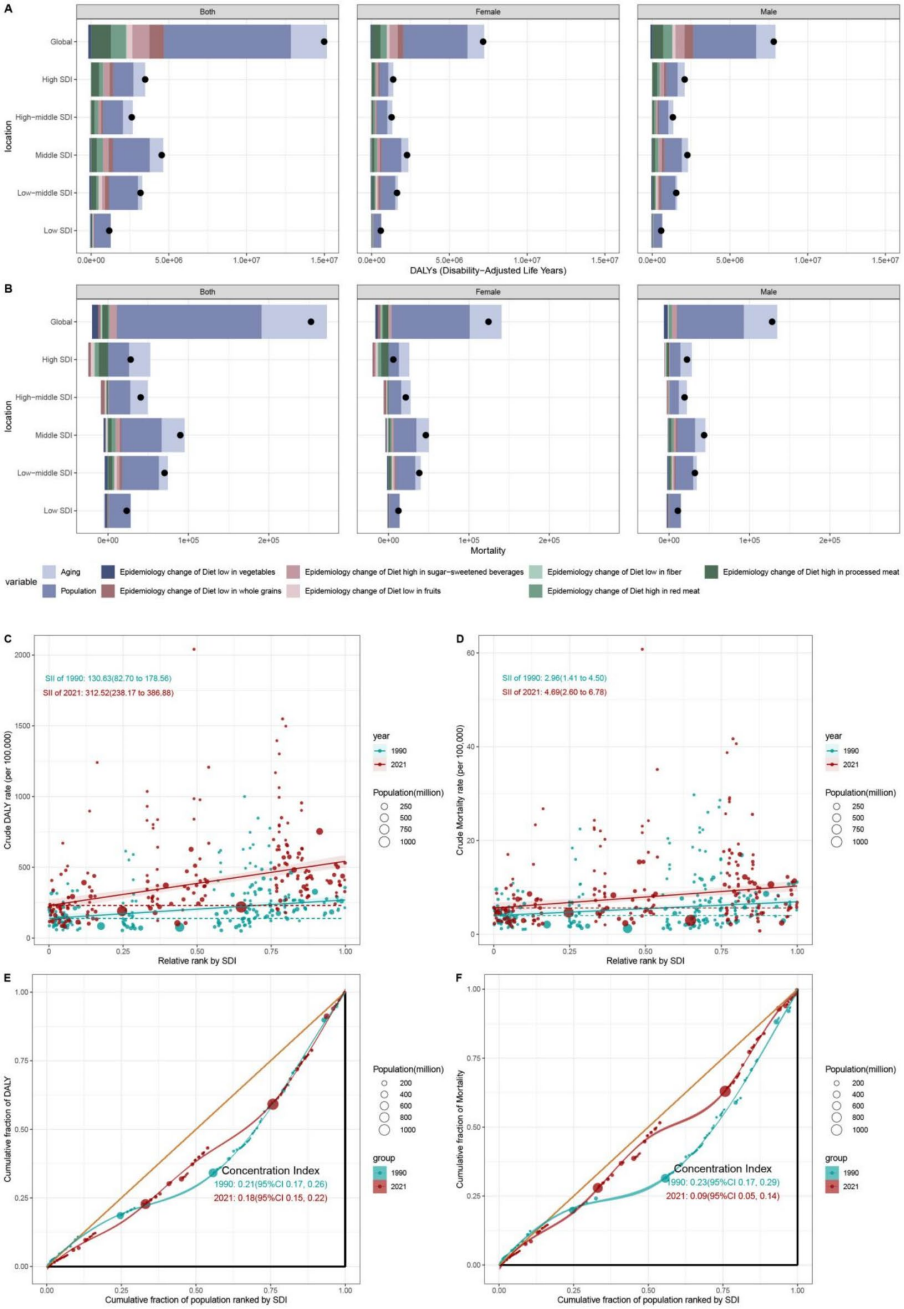
Decomposition and health inequality analysis of disability-adjusted life-years (DALYs) and mortality due to Type 2 Diabetes (T2D) attributed to dietary risk factors worldwide, 1990–2021. (**A**) Changes in global and Socio-Demographic Index (SDI) quintile-specific DALYs for T2D attributed to dietary risk factors. (**B**) Changes in global and SDI quintile-specific mortality rates for T2D attributed to dietary risk factors. Slope index of inequality (SII) (**C**) and Concentration Index (CI) (**E**) for DALYs due to T2D in 1990 and 2021, indicating health inequalities. Slope index of inequality (SII) (**D**) and Concentration Index (CI) (**F**) for mortality due to T2D in 1990 and 2021, indicating health inequalities.

The decomposition analysis of DALYs from 1990 to 2021 highlighted significant variations across socioeconomic and demographic groups (Fig. 6A, Table S7). Globally, population growth emerged as the primary factor, contributing 54.48% to DALYs, followed by aging at 15.48%. Among the dietary risk factors, the DALYs attributable to low vegetable intake were the only ones to show a marginal decline (− 1.19%). By contrast, high processed meat intake and sugar-sweetened beverage consumption emerged as the major contributors, accounting for 8.49% and 7.37% of T2D DALYs, respectively. In low SDI regions, population growth overwhelmingly influenced DALYs (91.44%), with aging and dietary changes negatively impacting by −4.30% and − 3.51%, respectively. Among females, aging (15.09%) and population growth (57.51%) were significant influencers of DALYs, with high processed meat and sugar-sweetened beverages intakes as primary dietary risk factors (7.93% and 7.27%). Males exhibited a similar pattern in terms of DALY influences.

In the analysis of global mortality trends from 1990 to 2021 (Fig. 6B, Table S8), aging and population growth emerged as the primary contributors, accounting for 32.20% and 71.34%, respectively. Epidemiological shifts in low vegetable intake and high processed meat intake slightly mitigated the overall difference (by − 3.08% and − 2.79%, respectively). Conversely, changes in high sugar-sweetened beverages and red meat contributed to an increase in mortality, by 3.56% and 0.70% respectively. High SDI regions were significantly affected by aging (94.38%), while low SDI regions experienced a surge in mortality due to population growth (118.16%). The influence of dietary risk factors varied across SDI quintiles. In high SDI regions, epidemiological changes of processed meat intake had the most substantial negative impact (−40.74%), whereas in low-middle SDI regions, these changes contributed to a positive effect (7.26%). Gender disparities were notable in high SDI regions: for males, aging and population growth contributed 64.29% and 58.95% to mortality, respectively, with changes in high sugar-sweetened beverages intake associated with a 3.90% increase, and changes in high processed meat intake linked to a (−12.54%) decrease. In contrast, females in high SDI regions experienced a more pronounced aging effect (220.25%), and significant contribution from population growth (216.71%). Changes in dietary risk factors consistently had negative impacts among females, with high processed meat intake (−150.81%) and high red meat intake (−66.88%) being the most substantial contributors.

### Cross-country health inequality analysis

The SII highlights a significant widening of disparities in T2D DALY and mortality rates between high and low SDI countries due to dietary risk factors (Fig. 6C and D). In 1990, the rates were 130.63 (95%CI 82.70–178.56) and 2.96 (95%CI 1.41–4.50) per 100,000, respectively, increasing to 312.52 (95%CI 238.17–386.88) and 4.69 (95%CI 2.60–6.78) per 100,000 in 2021. This trend underscores the disproportionate burden in nations with higher SDI, showcasing a growing gap over the years. Conversely, the CI, reflecting the gradient of inequality, decreased from 0.21 (95%CI 0.17–0.26) in 1990 to 0.18 (95%CI 0.15–0.22) in 2021 for DALYs, and from 0.23 (95%CI 0.17–0.29) to 0.09 (95%CI 0.05–0.14) for mortality (Fig. 6E and F). This shift indicates that the disparities in mortality burden among countries with varying socioeconomic levels are becoming more balanced.

## Discussion

Our study provided a comprehensive overview of the global impact of T2D attributable to dietary risks from 1990 to 2021. The study reveals a notable increase in both DALYs and deaths due to T2D worldwide, driven by dietary factors. The ASDR rose across genders, SDI categories, and regions. In contrast, the ASMR remained steady, even among males and in low SDI regions. Notably, among dietary risk factors, high processed meat consumption demonstrated the strongest association with rising ASDR and had the highest PAF; in contrast, the ASMR attributable to low vegetable intake exhibited the most pronounced decline, despite its comparatively lower PAF.

Our study identified a concerning trend in the global burden of T2D, with DALYs attributed to dietary risk factors increasing 1.92-fold and mortality rising 1.28-fold. This escalation is likely driven by factors such as population aging, growth, and modernization that promote obesity and sedentary lifestyles, creating a vicious cycle of increased T2D risk^33^. Our GBD analysis revealed significant variation in the contribution of dietary factors to the T2D burden. High consumption of processed meats and sugary beverages emerged as major drivers of increased T2D DALYs and mortality. This aligns with previous studies showing that high processed meat intake increases T2D risk by 12% and each additional daily serving of sugary beverages raises T2D risk by 26%^34^. Conversely, low vegetable intake was the only dietary factor linked to a decrease in ASDR attributed to T2D, likely due to the beneficial effects of fiber and micronutrients in vegetables on glucose control^35^. Decomposition analysis identified high processed meat and sugary beverage consumption as the primary dietary risk factors driving the increase in T2D DALYs, highlighting the potential for public health interventions targeting these specific factors^36–38^.

However, our study also highlights some differences. Unlike previous studies that focused primarily on individual dietary factors or specific regions, our analysis covers a broader range of dietary risks across 204 countries and 21 global regions. This comprehensive approach allows us to identify regional variations and global trends that may not be evident in more localized studies. For instance, while previous research has emphasized the benefits of high vegetable intake in reducing T2D risk, our study found an inverse association between low vegetable intake and ASMR, which may be influenced by regional dietary patterns or other confounding factors. This finding underscores the complexity of dietary influences on T2D and highlights the need for further investigation.

SDI serves as a key indicator for measuring a country’s level of development, deeply reflecting the inequalities faced by different countries and regions in the health sector. In high SDI countries, widespread exposure to processed foods, fast food culture, and sedentary work habits among residents has led to the prevalence of unhealthy lifestyles. This not only promotes the occurrence of chronic non-communicable diseases but also accelerates the growth of ASDR associated with T2D^39^. In low SDI countries, although ASMR remained relatively stable, the continuous increase in DALYs was primarily driven by population growth, highlighting the urgent need for effective preventive measures to curb the trend of T2D in resource-constrained regions^40^. The increasing SII further revealed that the gap in T2D DALYs and mortality rates between high SDI and low SDI countries was widening. This phenomenon underscores the exacerbation of global health inequality, possibly because high-income countries have more resources and advanced technologies for health promotion and disease management^41^. However, the decreasing CI indicated that the disparity in the burden of mortality was narrowing between countries with different socioeconomic levels. This may be attributed to the promotion of global health initiatives and improvements in primary healthcare services, which have enabled progress in reducing T2D mortality rates in low- and middle-income countries^42^. Addressing these challenges requires global cooperation and innovative strategies to ensure that all countries have access to the resources and support needed to improve health outcomes. This includes increasing investments in low SDI countries, enhancing the dissemination of health education, improving food environments, and promoting social movements for healthy lifestyles^43^.

Gender and age are two key influencing factors in the burden of T2D, closely associated with the occurrence, progression, and health outcomes of the disease. Our research findings indicated that ASDR of T2D in females was increasing at a slower pace compared to males, but ASMR in females had decreased. Possible reasons for this trend include differences in sex hormones, physiological variances, and variations in health behavior patterns. Studies suggest that estrogen in females may provide a certain degree of protection in the early stages, but as age advances, especially post-menopause, this protective effect diminishes, leading to an increased risk of T2D^44^. Furthermore, the incidence of T2D increases more rapidly in the elderly population, which is linked to the natural rise in insulin resistance among the elderly and the increasing prevalence of comorbid chronic diseases. This poses greater challenges for the elderly in managing T2D^45^. Additionally, population growth and aging are major factors contributing to the global increase in T2D DALYs, with a particularly significant impact on low SDI regions, underscoring the need for specific attention to prevention and early intervention measures in these areas. In high SDI regions, the impact of aging on T2D is more pronounced, necessitating enhanced health management for the elderly and chronic disease care services^46^.

A detailed regional analysis revealed significant disparities in the burden of T2D across different regions globally, reflecting the complex relationship between economic, cultural, environmental factors, and health. In Central Asia, rapid economic growth and urbanization have brought about lifestyle changes, including shifts in dietary habits. With the introduction of Western fast-food culture and the gradual disappearance of traditional dietary patterns, residents are consuming more high-energy, high-fat, and high-sugar foods. This dietary shift, combined with decreased levels of physical activity, has led to an increase in obesity rates, resulting in a significant rise in ASDR^47^. Additionally, environmental changes in the urbanization process, such as air pollution and increased life stress, may also have adverse effects on residents’ metabolic health^48^. Our research indicated a rapid increase in T2D ASMR in Eastern Europe, possibly linked to the dietary habits of the region’s inhabitants. Widespread consumption of high-fat and high-salt foods not only increases the risk of cardiovascular diseases but also contributes to the development of T2D. In Eastern Europe, high consumption of meat and salt in traditional dietary patterns, along with a rising consumption of processed foods, collectively promote the prevalence of T2D^49^. Moreover, uneven distribution of medical resources and inadequate chronic disease management services in some Eastern European countries may lead to poor treatment outcomes for T2D patients and an increase in mortality rates^50^.

### Limitations

While the GBD 2021 data offers valuable insights, it comes with inherent limitations common to GBD data. Observational studies, such as ours, are subject to several limitations that preclude the establishment of causality. First, confounding factors may influence the observed associations. For example, socioeconomic status, lifestyle behaviors (e.g., physical activity levels), and genetic predispositions could all impact the relationship between dietary factors and T2D risk. While we have attempted to control for some of these factors through stratification by Socio-Demographic Index (SDI) and other demographic variables, residual confounding may still be present. Second, reverse causation is another potential issue. For instance, individuals with existing metabolic conditions may alter their dietary habits, leading to spurious associations. Additionally, the accuracy of dietary intake data used in the GBD study may vary across regions, further complicating the interpretation of our results. Given these limitations, it is crucial to interpret our findings with caution. While our study highlights significant associations between high processed meat intake, sugary beverage consumption, and the burden of T2D, establishing causality would require more robust methodologies. Future research should focus on longitudinal cohort studies or randomized controlled trials to investigate the causal relationships between specific dietary factors and T2D incidence. Such studies can provide more definitive insights into the impact of dietary interventions on health outcomes.

### Practical implications of the findings

Our results highlight several key areas where targeted public health interventions could be effective in reducing the burden of T2D. First, the significant contribution of processed meat and sugary beverages to DALYs and mortality underscores the need for policies aimed at reducing their consumption. For example, public health campaigns could focus on educating communities about the health risks associated with these foods and promoting healthier alternatives. Additionally, policy measures such as taxation on sugary beverages or regulation of processed meat content could play a crucial role in reducing their consumption. These interventions have been shown to be effective in other public health contexts and could be adapted to address the rising burden of T2D. Despite the counterintuitive finding that low vegetable intake is associated with a reduction in ASMR, we emphasize the potential benefits of increasing vegetable consumption. Vegetables are rich in fiber, antioxidants, and other micronutrients that are known to improve glucose control and overall metabolic health. This finding may be influenced by regional dietary patterns or other confounding factors, and further research is needed to elucidate the underlying mechanisms. However, given the well-established benefits of vegetables in other studies, we suggest that public health initiatives should continue to promote vegetable intake as part of a balanced diet.

Our cross-country health inequality analysis highlights significant disparities in the T2D burden between high and low SDI countries. This underscores the urgent need for targeted interventions to address these inequalities. In low-resource settings, interventions should focus on improving access to healthy foods and promoting healthy lifestyles. This could include initiatives such as: (1) Ensuring that healthy foods are affordable and accessible in low-income communities. (2) Raising awareness about the importance of a balanced diet and regular physical activity. (3) Engaging local communities in the development and implementation of health promotion activities.

## Conclusions

Considering the comprehensive analysis above, the global burden of T2D continues to rise, closely linked to unhealthy dietary habits. Implementing multifaceted strategies, including improving the food environment, enhancing health education, and promoting healthy lifestyles, is crucial for reducing the incidence and mortality rates of T2D. Future research should further explore the heterogeneity of the relationship between dietary factors and T2D in different regions and populations, as well as how to more effectively implement and evaluate public health intervention measures.

## Electronic supplementary material

Below is the link to the electronic supplementary material.

Supplementary Material 1

Supplementary Material 2

## Data Availability

The datasets analyzed during the current study are available in the GBD 2021 online repository (http://ghdx.healthdata.org/gbd-results-tool).

## Abbreviations

AAPC: Average annual percentage change
APC: Annual percentage change
ASDR: Age-standardized disability-adjusted life-year rates
ASMR: Age-standardized mortality rates
ASR: Age-standardized rate
CI: Concentration index
CIs: Confidence intervals
DALYs: Disability-adjusted life years
GBD2021: Global Burden of Disease Study 2021
GATHER: Guidelines for Accurate and Transparent Health Estimates Reporting
GHDx: Global Health Data Exchange
IDF: International Diabetes Federation
PAF: Population Attributable Fractions
RR: Relative risks
SDI: Socio-demographic indices
SII: The slope index of inequality
T2D: Type 2 diabetes
