# Supplementary material for: Global regional, and national burden of type 2 diabetes attributable to dietary factors from 1990 to 2021
Source: Sci Rep. 2025 Apr 17;15:13278. doi: 10.1038/s41598-025-98022-y (PMC12006401; doi:10.1038/s41598-025-98022-y)
Supplement: Supplementary file 2 — Supplementary Material 2 [file 41598_2025_98022_MOESM2_ESM.docx]

**Supplementary Methods**

**Joinpoint regression analysis**

Time trend analysis is an important component of epidemiological research. Traditional regression models primarily fit and evaluate the overall trend of disease distribution within the study period from a global perspective, failing to capture local variation characteristics. In 1998, Kim et al. first proposed the Joinpoint regression model. The core idea of this model is to establish segmented regression based on the temporal characteristics of disease distribution. By dividing the study time into different intervals through several Joinpoints, the trend in each interval is fitted and optimized, allowing for a more detailed assessment of the specific disease change characteristics within different intervals of the overall time range[1]. The Joinpoint regression model, developed by the Division of Cancer Control and Population Sciences at the National Cancer Institute of the United States, has been widely applied in the field of trend studies on disease incidence and mortality rates.

(I) Model Introduction

The Joinpoint regression model includes two types: the linear model (y = xb) and the logarithmic linear model (ln y = xb). If the dependent variable follows a normal distribution (or approximately normal distribution) and the sample size is large (usually greater than 100), the linear model is preferred. For example, when the dependent variable is continuous variables like height, weight, etc. If the dependent variable follows an exponential distribution or a Poisson distribution, the logarithmic linear model is more suitable. For instance, when the dependent variable represents epidemiological data based on populations such as incidence rates, number of cases, etc. When analyzing trends in the incidence, prevalence, mortality rates, and DALYs rates of thalassemia based on population data, the logarithmic linear model is generally chosen.

(II) Modeling Method

The grid search method (GSM) is the default modeling approach used by Joinpoint. GSM divides the study data into a grid, with each grid intersection corresponding to a planned scenario[1]. Then, within the specified intervals, it computes performance metrics for the corresponding equations at each point using a fixed step size to determine the optimal function. In essence, the Joinpoint model uses the GSM to establish all possible segment function Joinpoints (i.e., Joinpoints) and calculates the sum of squares errors (SSE) and mean squared errors (MSE) for each possible scenario. It selects the grid point with the smallest MSE as the Joinpoint for the segment function and fits the equation parameters such as β_0_, β_1_, δ_1_, ..., δ_k_ based on the selected Joinpoints and interval functions[2].

(III) Model Optimization

Monte Carlo permutation test is the default model optimization method in Joinpoint software. Before modeling, it is necessary to set the range of the number of Joinpoints k as k ∈ (MIN，MAX) , where MIN represents the minimum number of Joinpoints, which is usually set to 0; MAX represents the maximum number of Joinpoints. Each permutation test checks the null hypothesis H_0_: the number of Joinpoints is k = k_a_, and the alternative hypothesis H_1_: the number of Joinpoints is k = k_b_. The permutation test starts from k_a_=MIN and k_b_=MAX. If H_0_ is rejected, k is set to k_a_ + 1 for further testing; if H_0_ is not rejected, k is set to k_b_ - 1 for another test, until k_a_ = k_b_, which means k = k_a_ = k_b_ is the preferred number of Joinpoints selected by the permutation test, and the corresponding model is the optimal model[3].

(IV) Index Calculation

Annual percent change (APC) and average annual percent change (AAPC) along with their 95% confidence intervals (CI) are the primary outcome indicators of the Joinpoint model. As the name suggests, APC represents the average annual percentage change of the dependent variable. For example, in a logarithmic linear model ln (y) =β_0_ + β_1_ x，where y represents the incidence rate and x represents the year of incidence, the formula for calculating APC in the fitted model can be derived as:


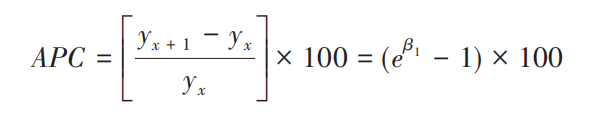


The lower and upper limits of the 100(1-α) % confidence interval are respectively:


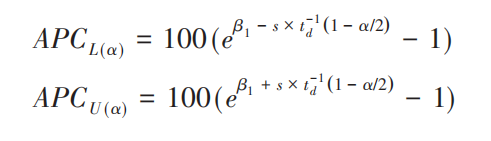


In the above formula, β_1_ represents the regression coefficient, s represents the standard error of β_1_, d represents the degrees of freedom, and t_d_(q) is the value corresponding to the qth percentile of the t-distribution with d degrees of freedom (such as 95%).

The APC is used to evaluate the internal trend of each independent interval of a segmented function or the overall trend with no connecting points. When it comes to assessing the overall average change trend encompassing multiple intervals, the AAPC is required. The parameter calculation method of AAPC involves weighted calculation of the regression coefficients of each interval based on the width w of the segment intervals. Its formula is as follows:


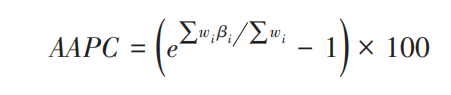


The lower and upper limits of the 100(1-α) % confidence interval are respectively:


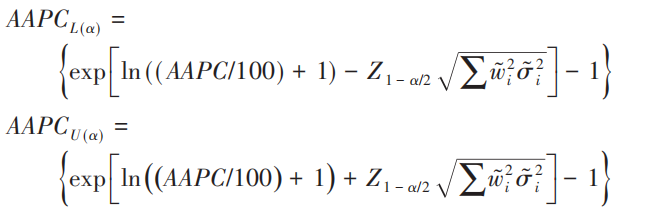


In the above formula, w_i_ represents the width of each segment function interval (i.e., the number of years included in the interval), β_i_ denotes the regression coefficient corresponding to each interval, σ^2^i is the variance of β_i_, and Z_α_ represents the corresponding value of the α percentile in the normal distribution.

(V) Software Download

To download Joinpoint software, we visited the website of the National Cancer Institute (https://surveillance.cancer.gov/Joinpoint/download), registered, and submitted our application information. Software citation: Joinpoint Regression Program, Version 4.9.1.0 - April 2022; Statistical Methodology and Applications Branch, Surveillance Research Program, National Cancer Institute.

**Decomposition analysis:**

We used the decomposition methodology of Das Gupta[4] to decompose disease`s DALYs and mortality by population age structure, population growth, and epidemiologic changes. For example, the number of DALYs at each location was obtained from the following formula:

DALY _ay, py, ey_ = $\sum_{i=1}^{20} ($a _i, y_ * p _y_ * e _i, y_)

Where DALY _ay, py, ey_ represented DALYs based on the factors of age structure, population, and DALYs rate for specific year y; a_i,y_ represents the proportion of population for the age category i of the 20 age categories in given year y; p_y_ represents the total population in given year y; and e_i, y_ represents DALYs rate given age category i in year y. The contribution of each factor to the change in DALYs from 1990 to 2021 was defined by the effect of one factor changing while the other factors were held constant. For example, the effect of age structure was calculated as:

[(DALY _a2021, p1990, e1990_ + DALY _a2021, p2021, e2021_)/3+ (DALY _a2021, p1990, e2021_ + DALY _a2021, p2021, e1990_)/6] - [(DALY _a1990, p2021, e2021_ + DALY _a1990, p1990, e1990_)/3+ (DALY _a1990, p2021b, e1990_ + DALY _a1990, p1990, e2021_)/6]

This method was applied similarly to assess the effects of population growth and epidemiologic changes. We calculated the percentage changes relative to the figures from 1990 as a baseline, thereby enhancing the comparability of the results across different SDI quintiles or gender groups within regions.

**References**

1. Kim HJ, Fay MP, Feuer EJ, Midthune DN. Permutation tests for joinpoint regression with applications to cancer rates. Stat Med. 2000; 19:335–51.

2. Kim S, Lee S, Choi J-I, Cho H. Binary genetic algorithm for optimal joinpoint detection: Application to cancer trend analysis. Stat Med. 2021; 40:799–822.

3. Yang JJ, Trucco EM, Buu A. A hybrid method of the sequential Monte Carlo and the Edgeworth expansion for computation of very small p-values in permutation tests. Stat Methods Med Res. 2019; 28:2937–51.

4. Chevan A, Sutherland M. Revisiting Das Gupta: refinement and extension of standardization and decomposition. Demography. 2009; 46:429–49.
